# Supplementary figures and images for: Activation of the ubiquitin-proteasome system contributes to oculopharyngeal muscular dystrophy through muscle atrophy
Source: PLoS Genet. 2022 Jan 13;18(1):e1010015. doi: 10.1371/journal.pgen.1010015 (PMC8791501; doi:10.1371/journal.pgen.1010015)

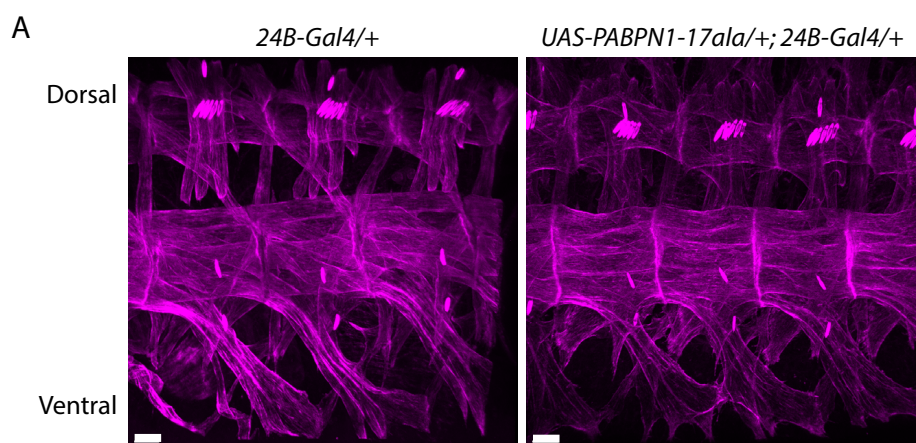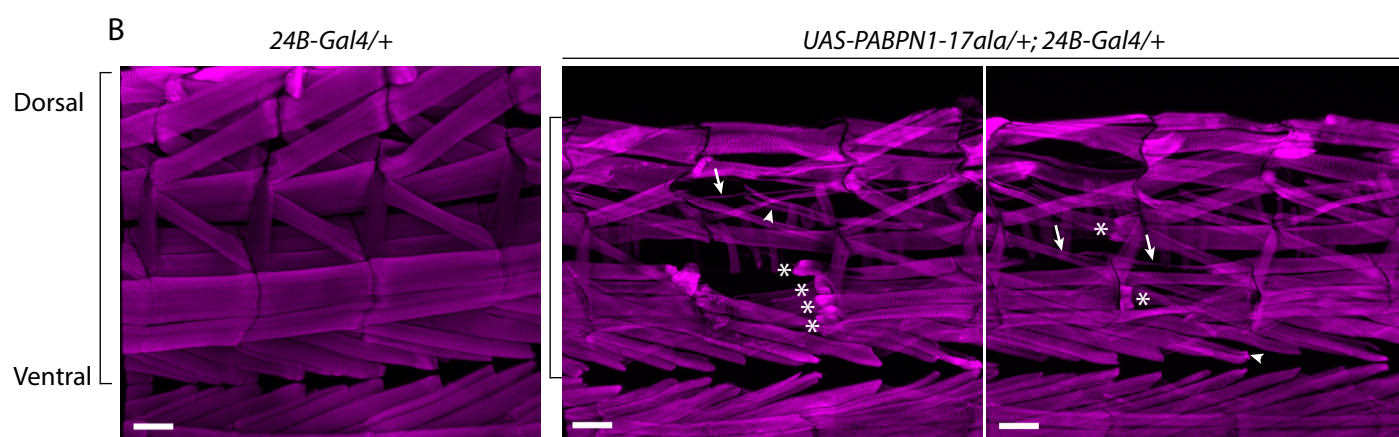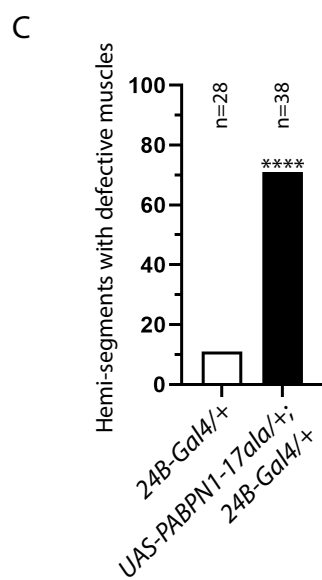

Figure S1

Supplement: S1 Fig — (A) Musculature of late PABPN1-17ala/+; 24B-Gal4/+ and control 24B-Gal4/+ embryos visualized using phalloidin staining. No major muscle defects were visible (n = 46 and 52 PABPN1-17ala/+; 24B-Gal4/+ and control embryos, respectively). Scale bar: 10 μm. (B) Musculature of PABPN1-17ala/+; 24B-Gal4/+ and control 24B-Gal4/+ third instar larvae visualized using phalloidin staining. Three adjacent hemi-segments are shown. Arrows point to very thin muscles, arrowheads to splitted muscles, and stars to broken muscles. All muscles in OPMD larvae are thiner than in control larvae. Scale bar: 100 μm. (C) Quantification of affected muscles visualized in B. The number of hemi-segments with defective muscle fibers described in B were scored. Note that in 24B-Gal4/+ control larvae, a single muscle fiber was defective per affected hemi-segment. ****p-value <0.0001 using the χ2 test. (PDF) [file pgen.1010015.s004.pdf]

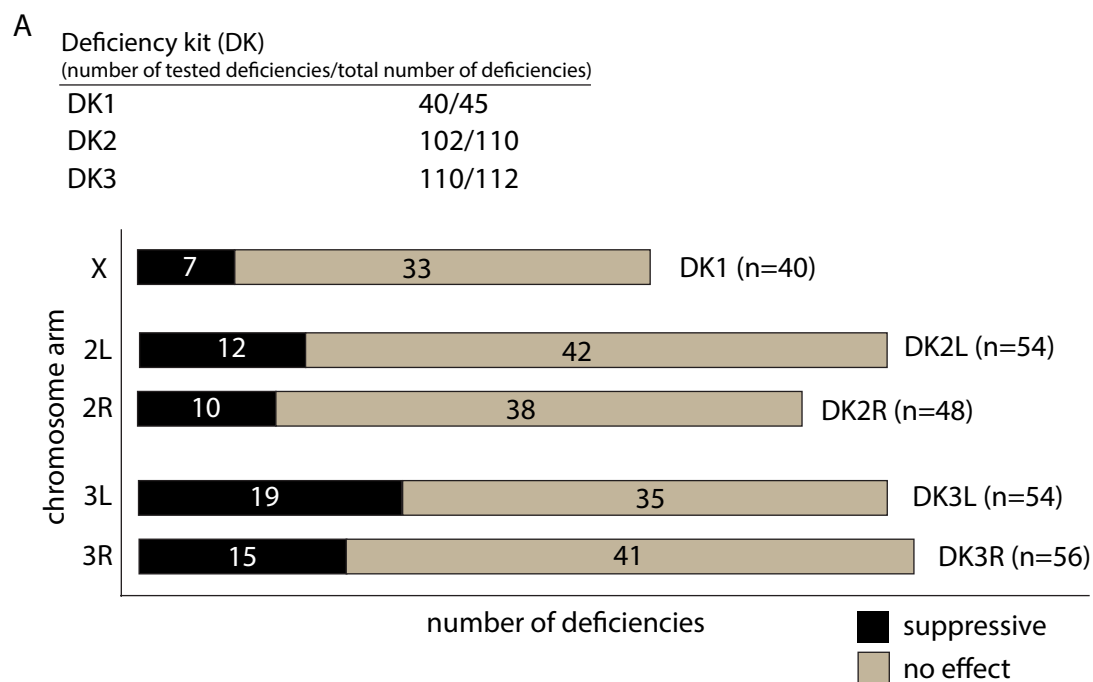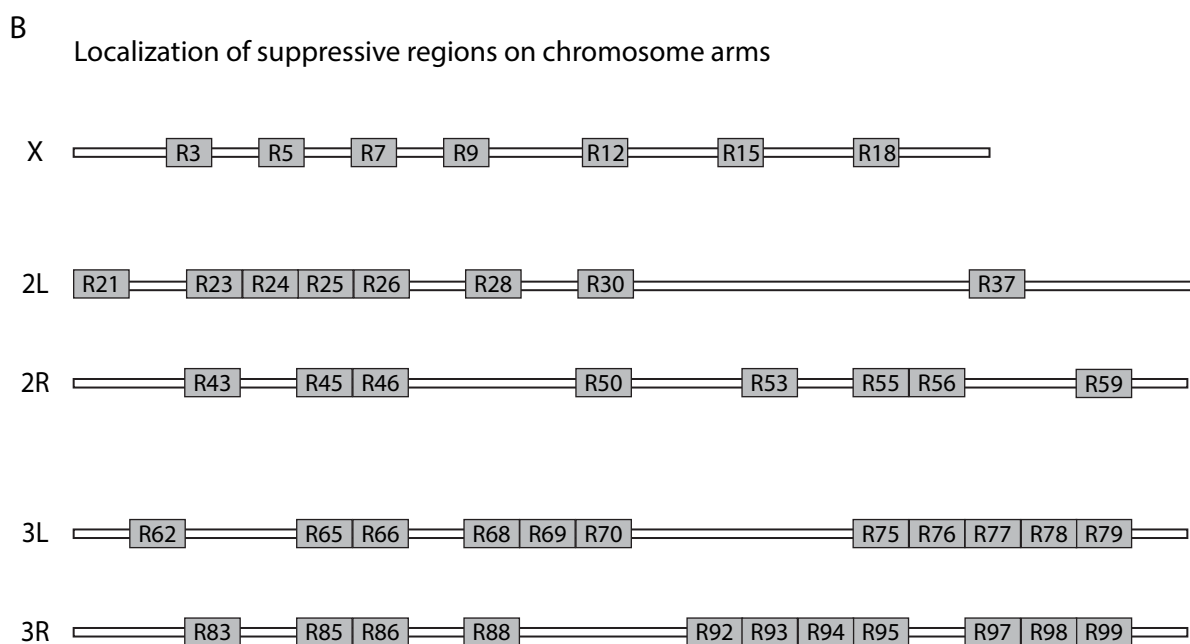

Figure S2

Supplement: S2 Fig — (A) Number of positive deficiencies from the Deficiency kit, identified in the genome-wide OPMD screen, per chromosome arm. The Deficiency kit from the Bloomington Drosophila Stock Center corresponds to a set of large deficiencies covering most of the Drosophila genome. (B) Localization of suppressive regions identified in the genome-wide OPMD screen, per chromosome arm. Suppressive regions were identified using large deficiencies from the Deficiency kit and smaller deficiencies spanning the large positive deficiencies. (PDF) [file pgen.1010015.s005.pdf]

A

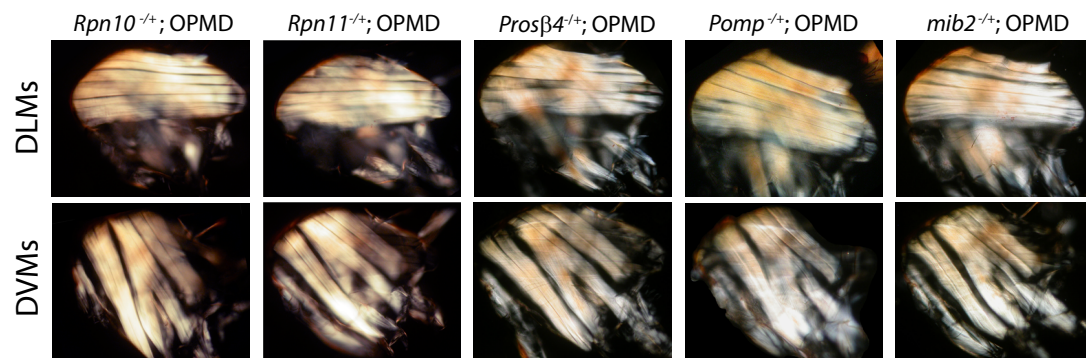

B

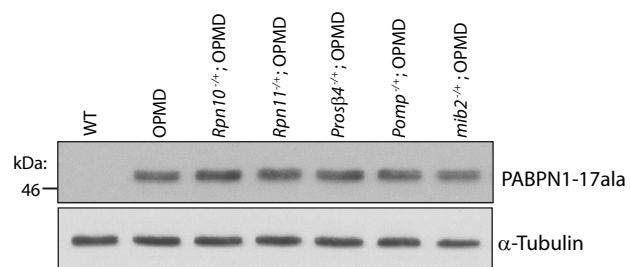

Figure S4

Supplement: S4 Fig — (A) IFMs in OPMD flies in the presence of UPS heterozygous mutants visualized under polarized light at day 11. IFMs are less affected in all genotypes compared to OPMD IFMs alone. Quantification of affected muscles is shown in Fig 3D. (B) Western blots of thoracic extracts revealed with anti-PABPN1 showing that the total amount of PABPN1 is not affected by mutants of the UPS components. α-Tubulin was used as a loading control. (PDF) [file pgen.1010015.s007.pdf]

A

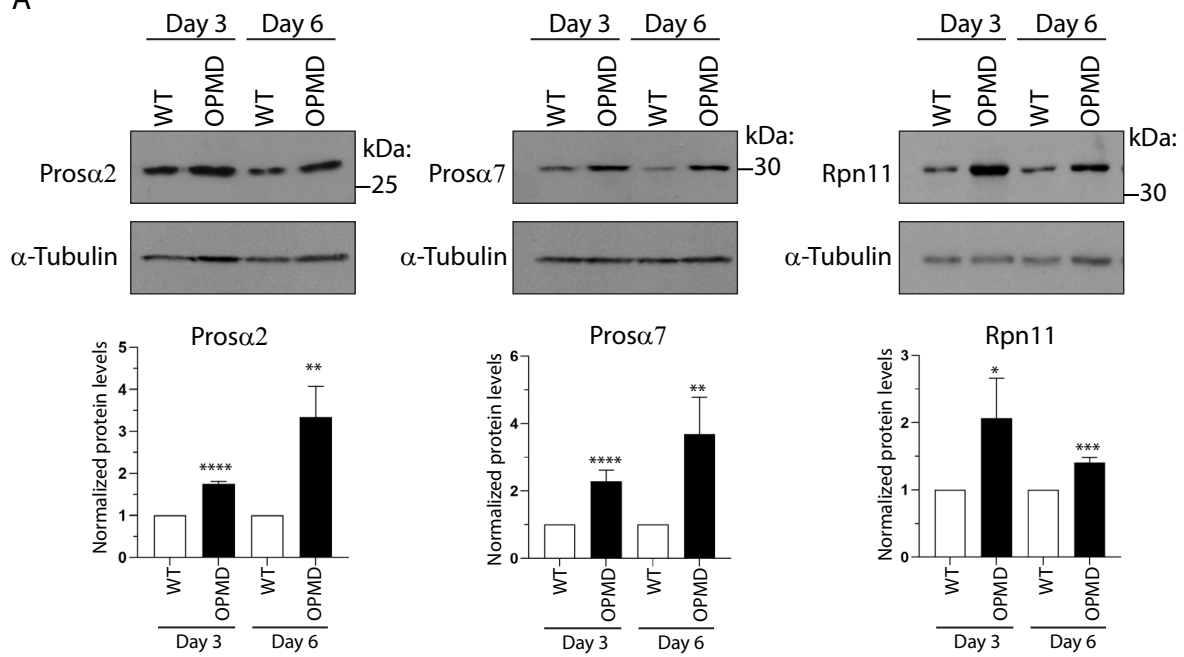

B

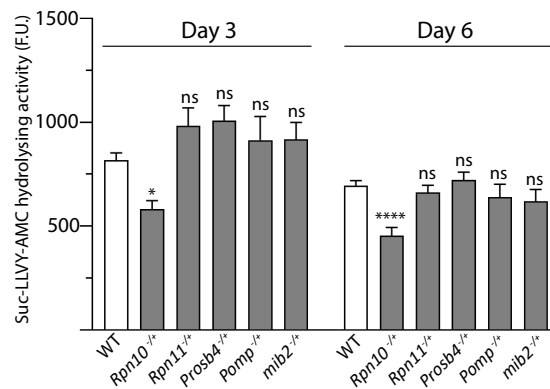

Figure S5

Supplement: S5 Fig — (A) Western blots of protein extracts from thoracic muscles of wild-type and OPMD (Act88F-PABPN1-17ala/+) flies at days 3 and 6 revealed with antibodies against three proteasome subunits: Prosα2, Prosα7 and Rpn11. α-Tubulin was used as a loading control. Quantification was performed using the ImageJ sofware with three to four biological replicates. Error bars represent SD. ****p-value <0.0001, ***p-value <0.001, **p-value <0.01, *p-value <0.05, using the unpaired Student’s t-test. (B) Proteasome chymotrypsin-like activity of protein extracts from wild-type and heterozygous UPS mutant thoracic muscles, at day 3 and day 6 of adulthood. Chymotrypsin-like activity was quantified by measuring AMC fluorescence following hydrolysis of Suc-LLVY-AMC. Means of three biological replicates quantified three times. Error bars represent SEM. ****p-value <0.0001, *p-value <0.05, ns: non significant, using the unpaired Student’s t-test. (PDF) [file pgen.1010015.s008.pdf]

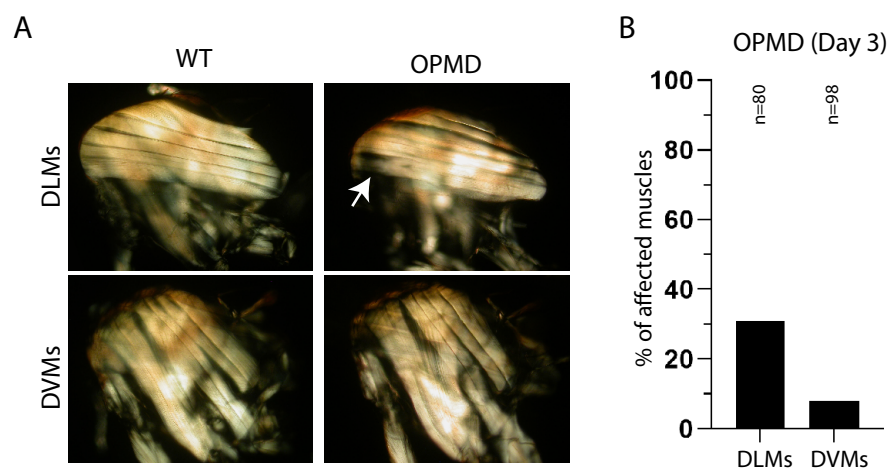

Figure S6

Supplement: S6 Fig — (A) IFMs in wild-type and Act88F-PABPN1-17ala/+ thoraxes visualized under polarized light at day 3. Six DLMs and seven DVMs were scored per hemi-thorax. The white arrow indicates a slight defect in a DLM, which is representative of the weak defects visible at that time. (B) Quantification of affected muscles. The numbers of scored DLMs or DVMs are indicated (n). No defects were observed in indirect flight muscles of wild-type flies (n = 54 DLMs and 105 DVMs). (PDF) [file pgen.1010015.s009.pdf]
