# Supplementary material for: Activation of the ubiquitin-proteasome system contributes to oculopharyngeal muscular dystrophy through muscle atrophy
Source: PLoS Genet. 2022 Jan 13;18(1):e1010015. doi: 10.1371/journal.pgen.1010015 (PMC8791501; doi:10.1371/journal.pgen.1010015)
Supplement: S3 Fig — (A) Schematic representation of UPS gene mutations used from Fig 3. The depicted mutations correspond to P-element or piggyBac insertions, except for mib21. Thin lines indicate intergenic regions and introns, open boxes represent UTRs and black boxes represent coding sequences. (B) Quantification of mRNA levels in the corresponding heterozygous mutant compared to wild type using RT-qPCR. RNAs were prepared from thoraxes of the indicated genotypes at day 6. sop was used as a control mRNA. Quantification in triplicates of three biological replicates. *p-value <0.05, ns: non significant, using the unpaired Student’s t-test. (C) Decrease of wing position defects following expression of Pomp RNAi in muscles with Mhc-Gal4. Left panel: Percentages of flies with abnormal wing position were scored at day 6; the numbers of scored flies are indicated (n). attP2 is the insertion site in which Pomp-RNAi was introduced and the stock bearing attP2 alone was used as control. **** p-value <0.0001, using the χ2 test. Right panel: quantification of Pomp mRNA levels using RT-qPCR. Legend is as in B. **p-value <0.01, using the unpaired Student’s t-test. (D) Effect of Prosβ1 heterozygous mutant in OPMD adult flies. Percentages of flies with abnormal wing position were scored at day 6; the numbers of scored flies are indicated (n). **** p-value <0.0001, using the χ2 test. Abbreviations and genotypes used in C and D are indicated. (PDF) [file pgen.1010015.s006.pdf]

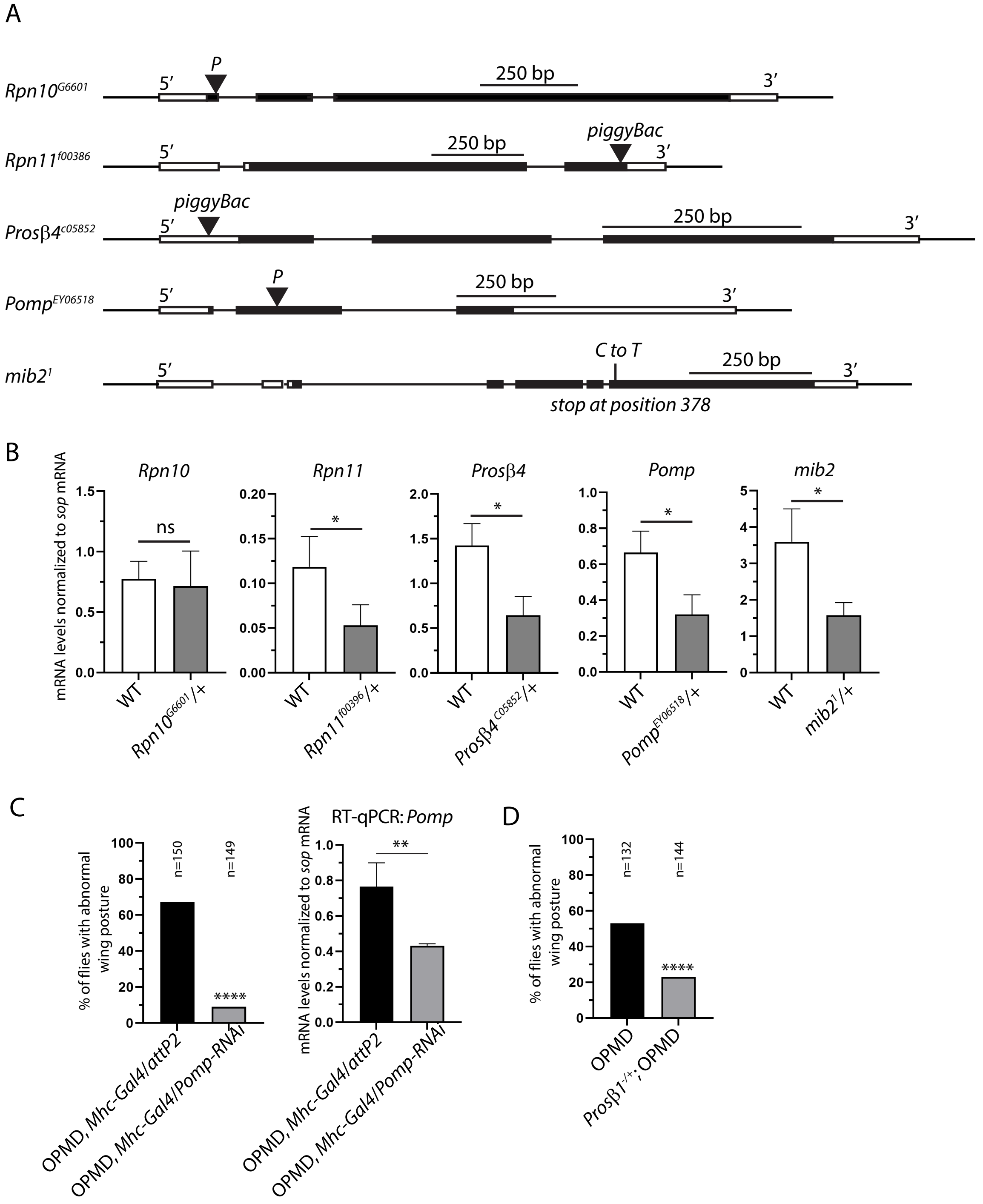

Abbreviations	
OPMD, *Mhc-Gal4/attP2*	*Act88F-PABPN1-17ala, Mhc-Gal4/P{caryP}attP2*
OPMD, *Mhc-Gal4/Pomp-RNAi*	*Act88F-PABPN1-17ala, Mhc-Gal4/Pomp*^{GLC01750}
OPMD	*Act88F-PABPN1-17ala/+*
Prosβ1$^{-/+}$; OPMD	*Prosβ1*^{05070}*/+; Act88F-PABPN1-17ala/+*

Figure S3
