# Supplementary material for: Activation of the ubiquitin-proteasome system contributes to oculopharyngeal muscular dystrophy through muscle atrophy
Source: PLoS Genet. 2022 Jan 13;18(1):e1010015. doi: 10.1371/journal.pgen.1010015 (PMC8791501; doi:10.1371/journal.pgen.1010015)
Supplement: Attachment — Submitted filename: Ribot et al Point-by-point Response.pdf [file pgen.1010015.s011.pdf]

Dr Martine Simonelig
mRNA Regulation and Development
Department Genetics and Development
Institute of Human Genetics, 141, rue de la Cardonille
34396 Montpellier Cedex 5. France
Tel: 334 34 35 99 59
e-mail: Martine.Simonelig@igh.cnrs.fr

PLOS Genetics Editors
PLOS Genetics

Manuscript number: PGENETICS-D-21-00502

Montpellier, November 25, 2021

Dear Dr Lu, Dear Dr Barsh,

I thank you very much for your decision regarding our manuscript number PGENETICS-D-21-00502, entitled "Activation of the ubiquitin-proteasome system contributes to oculopharyngeal muscular dystrophy through muscle atrophy".

We are very grateful to the reviewers for their thorough review of our manuscript and their insightful comments that prompted us to perform a series of new experiments. These new experiments are now included in the revised version of the manuscript, as additions to three figures and a Table (Fig. 3, Fig. 4, Fig. 8 and Table S2) and two new figures (Fig. S1 and Fig. S3). These new data strongly improve the manuscript. In particular, we have characterized the OPMD *Drosophila* model at embryonic and larval stages, and better characterized the model in adults through analysis of myofibrils and sarcomeric structure. In addition, we have used an *in vivo* assay to evaluate proteasome activity in ubiquitin-proteasome system (UPS) heterozygous mutants, an important point raised by the three reviewers. Thus, I am submitting a revised version of the manuscript in which we have addressed all comments of the reviewers.

The point-by point response to the reviewers is as follows.

Reviewer #1: In this manuscript, Ribot et al. examine the role that increased proteasome activity plays in a drosophila model of oculopharyngeal muscular dystrophy, which is due to polyA tract expansion in the PABPN1 nuclear protein. The authors designed a screen based on genomic deficiencies to identify modifiers of developmental lethality induced by expression of PABPN1 in skeletal muscle. They identify components of the ubiquitin proteasome as responsible for muscle degeneration induced by PABPN1. The authors went on to test the impact of heterozygous mutations for some components of the proteasome and associated proteins and found that they rescue muscle degeneration (as estimated with histological analyses and assessment of wing positioning). They also test whether MG132, a proteasome inhibitor, can reduce PABPN1-induced muscle degeneration and found it to be the case. The authors also report that preservation of muscle integrity occurs independently from PABPN1 aggregates, and suggest that proteasome hyperactivation (induced by mutant PABPN1) is the key reason for muscle degeneration.
Overall, this manuscript provides a sound take-home message given that while the proteasome is needed to degrade aggregation-prone proteins and for normal protein turnover, it is also known to cause muscle atrophy when overactive. Therefore it is not surprising that preventing its overt activation may be protective for muscle mass in certain conditions such as oculopharyngeal muscular dystrophy.
This reasonable take-home message is however not completely supported by the data included in this manuscript. There are a number of inconsistencies that weaken the manuscript and some assays do not appear robust, as explained in detail here below. In particular, most of the figures in the manuscript (Figure 3 onward) are based on the use of a series of heterozygous mutants for proteasome components

(Rpn10, Rpn11, ProsB4, Pomp) and for an E3 ligase (mib) which are used as tools to inhibit the proteasome. The authors find that in all cases these heterozygous mutants can rescue PABN1-induced muscle degeneration (Figure 3,5,6). However, whereas Rpn10 heterozygous mutants reduce proteasome activity (Fig. 4A), heterozygous mutants of Rpn11, ProsB4, Pomp, and mib do not affect the proteolytic activity of the proteasome (Fig. 4). On this basis, there is currently little evidence to conclude that proteasome inhibition is responsible for reducing muscle degeneration induced by PABPN1 as only Rpn10 heterozygous mutants seem to impact proteasome function. Moreover, it remains unknown how heterozygous mutations of Rpn11, ProsB4, Pomp, and mib2 rescue muscle degeneration via mechanisms unrelated from proteasome function.

We agree that accurately quantifying proteasome activity in heterozygous UPS mutants is a very important point and we performed new experiments to improve this quantification. First, it should be noted that mib2 is not a subunit of the proteasome and therefore proteasome activity is expected not to be affected in this mutant, as indicated in the manuscript p. 14. Second, *in vitro* quantification of the chymotrypsin-like activity used in Fig. 4A is not optimal to quantify proteasome activity *in vivo*. In particular, this assay is based on peptide degradation independently of protein ubiquitination. This point has now been clarified in the manuscript p. 14. Indeed in the *in vitro* assays, catalytic activity of the 20S proteasome is probed through degradation of peptides that diffuse into the catalytic chamber. In contrast, degradation of ubiquitinated proteins requires the 26S proteasome that includes additional catalytic activities able to process the substrat before injecting it to the catalytic chamber. Therefore, it is expected that mutants in different proteasome subunits behave differently between one another in *in vitro* assays, and also differently in *in vitro* assays and *in vivo*, depending on the affected subunit.

We now added an independent assay to measure proteasome activity *in vivo* and showed that proteasome activity is affected in all proteasome subunit (Rpn10, Rpn11, Prosβ4) mutants as well as in *Pomp* mutant (see below comment to Figures 3-4).

In addition, it is important to note that mutants at different steps of proteasome assembly and functioning (core proteasome, regulatory particle, proteasome chaperone) reduce OPMD muscle degeneration, making it very unlikely that the observed rescue could be mediated by yet undescribed mechanisms unrelated to proteasome function.

Specific comments:

Figures 1-2: these figures report schemes that summarize the results of the screen. It would be better to provide a summary with quantitative data, particularly for selected proteasome genes shown in Figure 2.

The quantitave data (% of pupae) are indicated in Tables 1 and 2 for each tested deficency and mutants. We have now added the number of scored pupae, as well as the results of several crosses with negative and positive controls in Table S2.

Figure 2: It is surprising to see several E3 ligases that rescue PABPN1-induced degeneration. E3 have normally specific set of target proteins and therefore normally have unique biological functions. Likewise, because DUBs can oppose E3 function by removing polyubiquitin chains, it is surprising to see that they score like E3s. It would be helpful if the author could show the quantitative data and provide information on the controls used in these experiments.

We don't have an experimentally validated explanation as to why several (in fact 4) E3 ligase mutant reduce OPMD muscle defects. We suppose that these E3 ligases are involved in the regulation of proteins important for muscle structure or function. Note that Diap2, an E3 ligase involved in apoptosis and innate immune response does not score positive in the screen.

Thank you for bringing the point about DUBs. In fact their biology is more complex and some of them can for example directly associate with E3 ligases, behaving as cofactors. We tested two DUBs in our experiments. One scored negative, and the other, *scny* scored positive in the screen. We found a recent publication that reported a role of *scny* in the regulation of Myc and an E3 ligase. Therefore, *scny* could play a role in OPMD through the regulation of these targets. We have now indicated this information in the manuscript p. 10.

We have added the number of scored pupae, in addition to their percentage, and the results of several crosses with negative and positive controls in Table S2.

Figures 3-4: The authors use a series of heterozygous mutants for proteasome components (Rpn10, Rpn11, ProsB4, Pomp) and an E3 ligase (mib) to test whether they can be used to inhibit the proteasome. They find that in all cases these heterozygous mutants can rescue PABN1-induced muscle degeneration (Figure 3).
However, whereas Rpn10 heterozygous mutants reduce proteasome activity (Fig. 4A), heterozygous mutations of Rpn11, ProsB4, Pomp, and mib do not affect the proteolytic activity of the proteasome (Fig. 4). On this basis, how do the authors explain the rescue of muscle degeneration by heterozygous mutations of Rpn11, ProsB4, Pomp, and mib2? There is currently little basis to conclude that proteasome inhibition is responsible for reducing muscle degeneration induced by PABPN1 as only Rpn10 heterozygous mutants seem to impact proteasome function.
Moreover, if not by impacting the proteasome, how would heterozygous mutations of Rpn11, ProsB4, Pomp, and mib rescue PABPN1-induced muscle degeneration?
As explained above (p. 2), mib2 is not a subunit of the proteasome. Proteasome activity is not expected to be affected in this mutant. This information is indicated in the manuscript p. 14. *mib2* heterozygous mutant would reduce muscle defects by decreasing the targeting of one or several proteins involved in muscle structure or function to the proteasome.

Because, *in vitro* quantification of the chymotrypsin-like activity used in Fig. 4A does not completely reflect the complex process leading to the degradation of a protein substrate by the proteasome *in vivo*, as discussed above (p. 2), we added an *in vivo* assay previously reported to evaluate proteasome activity (Low et al. BMC Cell Biology 2013). This assay is based on the formation of large aggregates containg p62 with ubiquitinated proteins, which arise upon KD of proteasome subunits. Using this assay, we found that heterozygous mutants of the three proteasome subunits, Rpn10, Rpn11, Prosβ4 and of *Pomp* have impaired proteasome activity. These new data are shown in Fig. 4C, D and described on p. 14.

The heterozygous mutants for proteasome components here used have not been characterized molecularly. What is the evidence that they reduce the mRNA or protein levels of the targeted genes (Rpn10, Rpn11, ProsB4, Pomp, and mib2)? qRT-PCR analysis should be sufficient to address this point.
Thank you for this remark. We have now clarified the molecular defects in these mutants in the new Fig. S3A, and performed RT-qPCR as requested. The results are shown in Fig. S3B. All mutants show reduced mRNA levels of the corresponding gene, except *Rpn10* mutant. This mutant corresponds to the insertion of a *P*-element in the first coding exon very close to the start codon and is likely a null allele, although mRNA levels are not decreased. This information is indicated on p. 11 of the manuscript.

The authors use whole-body heterozygous mutants for proteasome components. The conclusions would be strengthened by using transgenic modulation (such as RNAi) of these or other proteasome components targeted to skeletal muscle.
We used *Pomp* RNAi expressed with *Mhc-Gal4*, which was efficient in reducing *Pomp* mRNA levels in thoracic muscles, to show that *Pomp* suppressor effect occurred in muscles. These data are shown in Fig. S3C. It should be noted that many RNAi available from *Drosophila* stock centers cannot be used in our system, because the landing sites used to insert the RNAi constructs have already a suppressor effect on OPMD phenotypes. This is the case for the attP40 landing site of the TRIP RNAi from BDGP, and for the KK RNAi lines from VDRC.

Experiments with isogenic genetic background would be useful to avoid confounding effects deriving from genetic background mutations. At present, it seems that all experiments have been done with varied genetic backgrounds which could impact the outcome of these experiments.
Although we did not completely isogenize the mutants using five generations of crosses with w^{1118}, we changed the genetic background for the mutants *Rpn10*, *Rpn11* and *mib2*. This did not affect their capacity to reduce OPMD wing position defects. In addition, we used a different

mib2 allele, $mib2^{KG105081}$. This allele is hypomorphic based on its lethality at the third instar larval stage, whereas $mib2^{1}$ is lethal at the first instar larval stage. We found that $mib2^{KG105081}$ reduced OPMD wing position defects, but at a lower level than $mib2^{1}$, consistent with its hypomorphic nature. These results validate that the suppressor effects of the UPS mutants are due to mutations in UPS components and not to genetic background mutations. They are shown in the figure attached to this letter (Fig. 1 letter).

Figure 5: In this figure, the authors have scored the number of nuclei with PABPN1aggregates. They report that PABPN1 aggregates slightly increase in response to proteasome inhibition and on this basis argue that the aggregates per se are not the cause of muscle atrophy. While this reviewer agrees with this notion, the supporting data is weak.
Specifically, changes in the number of nuclei with aggregates as well as in the nuclear aggregate cross-sectional area appear minor, even if statistical significant (which is due to the high number of nuclei scored).
There is also some inconsistency across interventions: in Fig. 5D the nuclear aggregate cross-sectional area increases for Rpn10-/+, does not change for Prosb4-/+, and decreases for Pomp+/-. As these mutations are all supposed to affect the proteasome, it is puzzling on why they would score differently. But most importantly, why do the authors continue to use all these mutants? From studies in Fig. 4, they know that only Rpn10 mutants affect the proteolytic activity of the proteasome so I do not see any basis to employ other heterozygous mutants to inhibit the proteasome if in fact they do not.
There is inconsistencies in the impact of these proteasome mutations on "the percentage of nuclei with aggregates" (Fig. 5B) versus the "nuclear aggregate cross-sectional area" (Fig. 5D). One would expect these two estimates to be largely overlapping but they are not.
Based on the results in Fig. 4 that have shown an effect on proteasome activity only for Rpn10 mutations, I would have expected only Rpn10 mutations to score and that other mutants would have no effect. Instead Fig. 5B reports no effect of Rpn10-/+ on the percentage of nuclei with aggregates.
The authors should provide an explanation for these inconsistencies and use more robust (biochemical) methods to detect aggregate versus oligomeric PABPN1 if they want to draw conclusions on how inhibiting the proteasome affects PABPN1 aggregation.

The quantification of PABPN1 aggregates is a robust assay in our model to measure PABPN1 aggregation and we validated this assay in several publications (Chartier et al. 2006, 2009, 2015, Barbezier et al. 2011). Importantly, we thoroughly analyzed these aggregates including using electron microscopy, when we first described the *Drosophila* OPMD model (Chartier et al. EMBO J. 2006) and showed that they have the same structure as nuclear aggregates in patients. Note that we also previously tried to quantify PABPN1 aggregation biochemically using native protein gels but these assays were not conclusive.

In our previous studies, we found no correlation between the effect on the percentage of aggregates and the effect on their surface. In most cases only one of these measures is affected. For example, we showed that expression of the anti-PABPN1 nanobody reduces the surface of aggregates, but not their number (Chartier et al. HMG 2009). Similarly, feeding OPMD larvae and adults with the anti-aggregation drugs 6-aminophenanthridine and Guanabenz reduces the surface of aggregates, but not their number (Barbezier et al. EMBO MM 2011). Considering these data, it is not surprising that *Rnp10-/+* mutant increases the surface of aggregates without affecting their number (Fig. 5). In contrast, reducing the gene dosage of the *twin* gene that encodes the CCR4 deadenylase increases the number of aggregates, but not their surface (Chartier et al. PLOS Genetics 2015).

The different outcomes on PABPN1 aggregation between *Rpn10* and the other UPS mutants indicate differences in their mode of action. We propose in the manuscript that this could result from the specific role of Rpn10 in promoting 26S proteasome stability leading to stronger defects in proteasome activity and eventually increased PABPN1 aggregation in *Rpn10-/+* mutant. This is discussed on p.14 and in the legend of Fig. 8.

Comment: PABPN1 aggregation is described as a deleterious aspect. While that is certainly a disease biomarker that indicates loss of function of PABPN1, it has been shown in other contexts that it is

actually protective that aggregation-prone proteins are sequestered in defined compartments (aggregates or aggresomes), in order to reduce their interaction with native proteins. There are indeed several studies in other diseases showing that oligomers can be more toxic than larger aggregates.
Thank you for this comment that was also raised by reviewer #2. We have added this information in the Discussion p. 21.

Figure 6: same issues as above. The authors use heterozygous mutants for proteasome components that do not impact proteasome function (as demonstrated in Fig. 4) but they draw conclusions from these experiments as if these mutants would be inhibiting proteasome function.
New data in Fig. 4C, D show that proteasome activity is indeed impaired in mutants of the proteasome subunits and *pomp*. The increased levels of Mhc and Actin in the presence of *mib2*-/+ mutant would indicate either a direct role of this E3 ligase in targeting Mhc and Actin to the proteasome, or a more indirect role in regulating their degradation.

Figure 7: The authors show that feeding larvae with a proteasomal inhibitor (up to 600uM of MG132) reduces wing positioning defects due to mutant PABPN1. Why is this drug provided from larval development, what is the rationale for this? In fact, because there is no feeding through pupal development and because most larval muscle are histolyzed during pupal development, it is unlikely that larval feeding of MG132 has a direct impact in preserving myofibrils as these are degraded anyway during the pupal stages of development, apart for persistent muscles. Moreover the PABPN1 transgene seems to be expressed under the control of Act88F, which is not expressed in larval muscles. On this basis, it is unclear how feeding MG132 at the larval stages will impact aggregation and toxicity of PABPN1 in adult muscles at day 1. What is the reason of this larval feeding regimen? Are the same results obtained with MG132 feeding restricted to adult flies?
We have tried previously to measure a positive effect of MG132 using adult feeding only and we tried again during this revision. However, adult feeding does not lead to reduced wing position defects. The effect of the drug depends on its bioavailability in muscles, which is difficult to predict. Because larvae feed a lot and MG132 is a stable compound, we suppose that a certain amount of drug is still present in the adult following larval feeding. The regimen we used is larval <u>and</u> adult feeding at the same concentration of drug up to the day of scoring wing position. We start scoring wing position at day 3 of adulthood. This information is indicated in the Result section on p. 17 and in the Materials and Methods on p. 23.

Reviewer #2: The authors use a Drosophila model of oculopharyngeal muscular dystrophy (OPMD), made by expression of a mutant mammalian poly(A) binding protein nuclear 1 (PABPN1) protein containing an expanded polyalanine tract, to follow up on previous transcriptomic work showing a deregulated ubiquitin-proteasome system (UPS). OPMD is a complex muscle disease, wherein multiple functions are affected due to the presence of the mutant protein. Here the authors focus on protein degradation and PABPN1 aggregation. They used both targeted and genome-wide genetic screening for improved muscle function to identify specific UPS components whose downregulation reduces myofibrillar protein degradation. These do not consistently reduce PABPN1 aggregation. This contrasts with previous work showing that reducing PABPN1 aggregation improves muscle function. Further, inhibition of the proteasome yields similar results, suggesting a potential therapeutic approach for disease treatment. This is a well-done paper containing a tremendous amount of experimental data, with a strong genetic component. The following comments should be considered by the authors:

1. It would be helpful to provide more rationale for the Drosophila model. Why is the mammalian mutant PABPN used instead of a mutant Drosophila protein? Where is the mutant gene expressed and by what methodology? This may have been established in previous papers, but is important enough an issue to mention here. Further, it would be useful to point out that while it is a hybrid model, the screen may prove valuable for therapeutic use in that it is actually testing how defects affiliated with the mammalian protein can be suppressed.
Thank you for this comment. We have now clarified these points on p. 7 of the manuscript.

We have set up the model by expressing the mammalian mutant PABPN1 because the disease is dominant and due to expression of this mutant protein. Thus, we believed this would produce the more accurate model. In addition, the *Drosophila* protein does not have a polyalanine tract at its N terminus.

2. While the genome-wide screen seems extremely effective in identifying suppressors, the fact that the suppressors were in previously identified pathways is not particularly surprising. This is because the investigators tested candidate genes (at least partially) based upon those with reported relationships with OPMD and/or PABPN1. This should be pointed out in the paper.
We have added this comment on p. 9.

3. The finding that reduction in expression of 77% of tested UPS genes suppressed lethality was impressive. Do the authors care to speculate why the remaining 23% did not? In this regard, the IFM defect suppression experiment is quite convincing, but did the authors try a negative control, i.e., a mutant for one of the UPS components that did not suppress lethality in the directed screen?
Prompted by this comment, we chose the *Prosβ1* mutant that scored negative in the larval lethality screen (Fig. 2A) and tested it in adults. We found that this mutant was able to reduce wing posture defects in the adult. This indicates that the number of suppressor genes was yet underestimated with the larval lethality screen. This information was added on p. 11 and in Fig. S3D. It is expected that a number of mutants should behave differently on OPMD phenotypes in larvae and adults, due for example to the nature of mutants (mutants affecting the gene in only one of these two stages).

4. For the western blots in Fig. 3E and Fig S2, how were the samples prepared? Presumably not by the myofibrillar protein isolation procedure given in the Methods section. For all western blots: are the blots for the control protein from the same gel or at least the same samples? This should be stated.
We have added the information about protein preparation for regular western blots and the fact that the same blots were used to analyze the loading in Materials and Methods p. 24.

5. Please speculate in the text on how the mutants work to reduce proteasome function, if activity of the proteasome is maintained in most of them (Fig. 4)?
As explained above in response to the general comment (p. 2) and comment on Figures 3-4 (p. 3) from reviewer #1, *in vitro* quantification of the proteasome activity based on the chymotrypsin-like activity does not perfectly evaluate proteasome activity *in vivo*. Therefore, we used an *in vivo* assay to evaluate proteasome activity. Using this assay, that corresponds to the formation of large aggregates containg p62 with ubiquitinated proteins, we found that heterozygous mutants of the three proteasome subunits, Rpn10, Rpn11, Prosβ4 and of *Pomp* have impaired proteasome activity. These new data are shown in Fig. 4C, D and described on p. 14.

6. It would be better to state in the abstracts and elsewhere that the improved muscle structure/function observed due to reducing UPS components does not correlate with the levels of aggregate accumulation, since some of the current wording implies there is no change in aggregate accumulation.
We have changed the wording accordingly in the abstact and in the subheading p. 15

7. Why not include the statistical significance indicators in Fig. 5C?
We have added the statistical data in Fig. 5C.

8. Why was tubulin replaced with a different control protein in Fig. 6E?
The Tubulin showed some variability on these gels with the UPS mutants, thus we used a ribosomal protein as an independent loading control.

9. It would be helpful to show (or refer to from another publication) a confocal or TEM image of sarcomeres from PABPN1 muscles and from PABPN1 muscles that have been rescued. Is there

sarcomere degradation that is prevented?
We have thoroughly addressed this point using immunostaining with sarcomere components and confocal miscroscopy. These new data are shown in Fig. 3E, F, described on p. 12 and commented in the Discussion p. 18. Indeed, sarcomere degradation is prevented with the mutants.

10. The authors might mention that aggregates could serve a useful function in removing misfolded proteins from the soluble cytoplasm to prevent their aberrant interactions.
Thank you for this comment, we have added it in the Discussion p. 21.

11. A number of the statistical comparisons (e.g., Fig 7C-E) compare only two independent experiments. Does PLoS permit this?
We have not found restrictions regarding this point in PLOS Genetics policies.

Numerical data are not provided for a few figure panels, just summary statistics and plots.
We have provided the numerical data in Table S3.

Reviewer #3: This ms submitted by C. Ribot et al contains data describing the contribution of increased proteasome activity to oculopharyngeal muscular dystrophy (OPMD). The data are based on an *in vivo*, induced model of OPMD in *Drosophila*, with flight muscles as main model tissue. Specifically, the authors propose that increased proteasome activity, subsequent to (forced) expression and nuclear aggregation of PABPN1-17ala, an OPMD signature, is associated with proteosomal degradation of myofibrillar proteins. This in turn could be causal to muscle defects. Feeding *Drosophila* larvae with a proteasome inhibitor at sub-lethal levels does reduce muscular defects, opening the possibility of future pharmacological treatment of OPMD.
OPMD, a progressive human disease differentially affecting specific sets of muscles is thought to result from accumulation of extended-polyalanine forms of the nuclear PABP PABPN1 (PABPN-11-18ala). Transcriptome analyses previously performed in different models pointed to an increased expression of components of the ubiquitin-proteasome (UPS) protein degradation pathway. The potential involvement of UPS in progression of OPMD has since been an important question, in parallel to mitochondrial dysfunction. Here, the authors address, for the first time in vivo, the consequences of UPS up-regulation using Drosophila flight muscles as model. The starting point is an unbiased genetic screen for suppressors of PABPN17ala-induced lethality, from which the UPS pathway emerges as one candidate pathway (Fig.1-2). The authors assay further the consequences of decreased proteasome activity on induced OPMD, by genetically assaying mutations in 5 individual UPS components. The data show that reduced proteasome activity correlates with reduced PABPN17ala-induced adult muscle defects (Fig 3,4). The rescue does not *consistently* correlate with reduced levels of PABPN1 aggregation, however, although the variability in assays (Fig.5) could prevent firm conclusions (see also Fig6). It correlates with rescue of Mhc and actin levels, compared to OPMD mutants (Fig.6). The authors thus conclude that one consequence of UPS deregulation in PABPN1-17ala mutants is an increased degradation of myofibrillar proteins which could be the molecular basis of OPMD.
An in vivo analysis of the relation between UPS deregulation and OPMD, going from a genetic screen to myofibillar defects, is of interest to a wide range of colleagues. Yet, the data shown are mostly correlative, except for genetic analyses, and rather patchy experiments do not allow strong conclusions. This is well reflected by the general tone of the manuscript with reiteration of “significant” “substantial”, “tendency” terms in author’s conclusions. Below are some specific suggestions for improving the manuscript.
In this manuscript we are using the genetic approach to functionally address the role of the UPS in OPMD. In Fig. 3 and 6, we show that myofibril disorganization and the loss of sarcomeric structure (Fig. 3) together with the loss of myofibrillar proteins (Fig. 6) are reduced in the presence of UPS mutants that lead to partial rescue of wing position defects. We believe that these data provide strong support to our conclusion that the UPS acts in OPMD through degradation of myofibrillar proteins.

We do not understand the semantic comment on the utilization of the terms “significant” and “substantial”. "significantly" is used in the manuscript to mention that the

differences are statistically significant. "substantially" is used once in the manuscript, on p. 14 to mention the strong effect of increased proteasome content and activity in OPMD. "tendency" is also used once in the manuscript, on p. 16.

Comments/suggestions

Introduction versus discussion

A large part of the introduction recalls diverse molecular consequences of alanine triplet expansion in PAPBPN1, a mutation which leads to PABPN1 aggregates in muscle fibers. The question is then how PABPN1 aggregates lead to progressive weakness of specific muscles. The present ms does little answer this question and ignores the progressive character of the human disease. Many interesting points raised in the introduction need to be better looked at, in the discussion in view of the present data.

Thank you for this remark. We have now added information regarding the progressivity of the defects in our models (p. 8, 12) and discussed our results with regard to the disease progressivity on p. 20. We have also expanded the Discussion p.18 and 21.

Fig. 1. Lethality screen

The screen is based on lethality induced by 24B >PABPN1-17ala, scored in pupae. Although this scoring is adequate for an initial, unbiased screen, a more precise description of the period of lethality and muscle phenotype induced by PABPN1-17ala mesodermal expression should be given. Looking at muscle pattern defects in embryos or larvae, could inform on the selectivity and progression of muscle defects with exercise, and better connect to the rest of the ms (IFM defects).

We agree that this is an important point. We have now characterized the *24B-Gal4 >PABPN1-17ala* model in late embryos and larvae using muscle phalloidin staining. These new data are shown in Fig. S1 and described on p. 8.

Fig.3 The adult muscles phenotype

In previous studies, the authors used a muscle specific driver, MhcGal4 (Chartier et al., 2006, 2015). Using this driver, *muscle defects were clear by day 6 and increasing with time to reach 100% of the thoraxes at day 16 (Chartier et al., 2006).* In this ms, Ribot et al., use Act88FGal4 (ubiquitous expression?) to drive PABPN1-17ala expression in adult IFMs. The fraction of muscle defects (scored at day 11) seems lower. Could the authors comment why they changed driver.

We used the construct Act88F-PABPN1-17ala to express PABPN1-17ala specifically in adult indirect flight muscles. This information is indicated on p.11. We already developed and used this model in Chartier et al. PLOS Genetics 2015. This model presents several advantages as follows. 1) Expression is independent of Gal4, this simplifies the genetics (one construct instead of two) to introduce other genetic components. 2) Crosses are performed at 25°C instead of 18°C for the *Mhc-Gal4>UASPABPN1-17ala* model, which is more convenient and reduce the time to obtain the model flies. 3) The phenotypes are weaker than that of *Mhc-Gal4>UASPABPN1-17ala* flies, which is more adapted to screen for both suppressors and enhancers of the phenotypes.

Referring to the percentage of muscle defects is very vague and reference to publication [40] for the analysis of these defects seems excessive (see Fig.2 in ref 40, for comparison). As it is, Fig. 4C, the only tissue level analysis within this ms, is not informative. Please indicate the type of defects which were recognized, how muscles were scored as altered, and the changes which occur in either “OPMD” or/and rescued flies between day 6 and 11. It would be a real plus to introduce a sarcomeric marker (see for example Sarov et al., 2016; PMID: 26896675) in order to compare the OPMD phenotype to « myofibrillar genes » phenotypes (Schnorrer et al. ? 2010 PMID: **2022084).** As mentioned for Fig.1, the progressivity, or not, and specificity of the myofibrillar defects deserves more attention.

We agree that this is a very important point and accordingly we performed the requested experiments. We thoroughly analyzed muscle defects using immunostaining to visualize Mhc and Kettin. These data are shown in Fig. 3E, F and described on p. 12. We think that this is an important addition to the manuscript.

We also detailed, as requested, the defects visualized using polarized light in Fig. 3C.

Fig.4 Proteasome activity
Analysis of proteasome activity is at days 3 and 6 (any change at day 11?) reveals higher activity in OPMD compared to wild type (or Rpn10-rescued) adults, consistent with previous transcriptome analyses. No return to wt proteasome activity levels was observed, however, in transheterozygous flies for 4 of 5 UPS mutants, although rescue of flight muscle/wing posture was effective and similar to Rpn10 (Fig. 3B,3D). Therefore, bases on these sole data, is it legitimate to conclude that *OPMD is associated with increased proteosomal content and activity in muscles that substantially contributes to OPMD defects?* Can one exclude that Rpn10 could play other functions?. The lack of consistent correlation does not support fully the conclusion and weakens the rest of the ms.
This is an important point that was also raised by reviewers #1 and #2. As explained above (p. 2, 3 and 6) in response to comments from reviewers #1 and #2, *in vitro* quantification of proteasome activity based on the chymotrypsin-like activity does not accurately evaluate proteasome activity *in vivo*. Therefore, we used an *in vivo* assay (described in Low et al. BMC Cell Biology 2013) to evaluate proteasome activity. Using this assay that quantifies the formation of large aggregates containg the p62 protein with ubiquitinated proteins, we found that heterozygous mutants of the three proteasome subunits, Rpn10, Rpn11, Prosβ4 and of *Pomp* have impaired proteasome activity. *mib2* does not encodes a proteasome subunit, therefore *mib2* mutant is not expected to show lower proteasome activity. Its postitive effect on OPMD defects would be through its function in ubiquitination of proteins involved in muscle structure or function. These new data are shown in Fig. 4C, D and described on p. 14.

As discussed above in response to reviewer #1 general comment on p. 2, the fact that mutants at different steps of proteasome assembly and functioning do reduce OPMD muscle degeneration, makes it unlikely that this effect, including in *Rpn10* mutant, might be mediated by mechanisms independent of proteasome function and yet to described.

Fig.5 Reduced OPMD muscle defects by UPS mutants do not correlate with reduced PABPN1aggregation.
The authors observe a correlation between decreased amounts of PABPN1 aggregates and rescue of muscle defects in 3 proteasome mutants (Rpn11, ProsB4, Pomp). Rpn10 mutants again behaves differently than other UPS mutants, with more aggregates, correlating well with a decreased proteasome activity, as underlined by the authors. Taking into account Fig.4, it seems that the data support the conclusion that rescue of muscle defects does not correlate well with increased proteasome activity, while PABPN1 degradation does (although see Fig.7). The author's conclusion that « *these data show that the reduction of muscle degeneration in the presence of UPS mutants does not consistently result from reduced PABPN1 aggregation* » is valid but fairly incomplete.
New data in Fig. 4C, D show that proteasome activity is impaired in mutants of the three proteasome subunits (Rpn10, Rpn11 and Prosβ4) and in *Pomp* mutant. Nonetheless, it it true that the reduced chymotrypsin-like activity recorded in the *Rpn10*-/+ mutant correlates with the specific behavior of this mutant in increasing PABPN1 aggregation. This indicates differences between Rpn10 mode of action and that of other proteasome subunits, as discussed above. We propose in the manuscript that this could result from the specific role of Rpn10 in promoting 26S proteasome stability leading to stronger defects in proteasome activity and eventually increased PABPN1 aggregation in *Rpn10*-/+ mutant. This point is discussed on p.14 and in the legend of Fig. 8.
We have changed the wording of the conclusion on p. 16.

Fig.6 Myofibrillar proteins are more ubiquitinated and degraded in OPMD muscles.
The authors show here that myofibrillar proteins (here, Actin and Mhc) are more ubiquinated and their amounts reduced in OPMD flies. Increased ubiquitination and clearance is already observed at day 3. How do the authors interpret that this reduction does not increase with time, while muscle defects do ? The myofibrillar proteins amount is restored (strong tendency to --) in some UPS mutant rescued flies (Fig.6E), without, again, consistent correlation with rescue of proteasome activity (Fig.4A, C) or rescue of muscle defects at day 11, Fig. 3D. In view of these results, is it appropriate to conclude that the involvement of the UPS of OPMD pathogenesis depends of its function in the degradation of

myofibrillar proteins ?. This may well be true but the data shown are only correlative, and authors need to be more cautious.

Clearance of Actin and Mhc might increase with time. The quantification of western blots is not that precise that it would allow to record variations if they are not very strong. In addition, we do not think that muscle defects result from decreased amounts of two proteins only, but that they are rather due to affected levels of many proteins involved in muscle structure. Mhc and Actin were used as examples of myofibrillar proteins. This might also be an explanation of the fact that several E3 ligases scored positive in the screen. Muscle defects and their progressivity would result from increased clearance of many muscle proteins that would build up with time. Thus, we do not expect a strict numerical correlation between the levels of Actin and Mhc in UPS mutants and the capacity of these mutants to reduce muscle defects.

As explained above, we have now shown using an *in vivo* assay that proteasome activity is reduced in mutants of the three proteasome subunit and in *Pomp* mutant.

We have changed the wording of the conclusion p. 17.

Fig.7 Pharmacological inhibition of proteasomal activity reduces OPMD muscle defects

The authors choose the highest non toxic concentration (600 micoM) of the proteasomal activity inhibitor MG132 to further assay the role of UPS in OPMD, using wing posture as read-out. Interestingly, “significant” rescue is similarly observed with 400 microM or 500 microM concentrations (Fig. 7B). PABPN1 aggregation is not decreased. These very promising data are consistent with genetic analysis of one UPS mutant, Rpn10, but do not fully explain wing posture rescue by other UPS mutants (Fig.3) which remains very puzzling.

As indicated above, new data in Fig. 4C, D show that proteasome activity is impaired in mutants of the three proteasome subunits and in *Pomp* mutant.

Fig.8

It is worth underlining the new data (in red or bold characters, rather than underlining previous data) and leaving hypothetical connections in italics (for example, degradation (inhibition?) of the PABPN1 oligomer), to make the figure more self-explanatory. If Rpn10 and MG132 to be mentioned in the figure legend, they need to appear on the figure. Muscle atrophy is not what is described here (Fig.4)

We have modified Fig. 8 accordingly. The new Fig. 3E and S1B show muscle atrophy.

We believe that this revised version that includes a large amount of new data is highly strengthened and we hope that you will find it satisfactory.

I thank you very much in advance and I am looking forward to hearing from you.

Sincerely yours.

Martine Simonelig

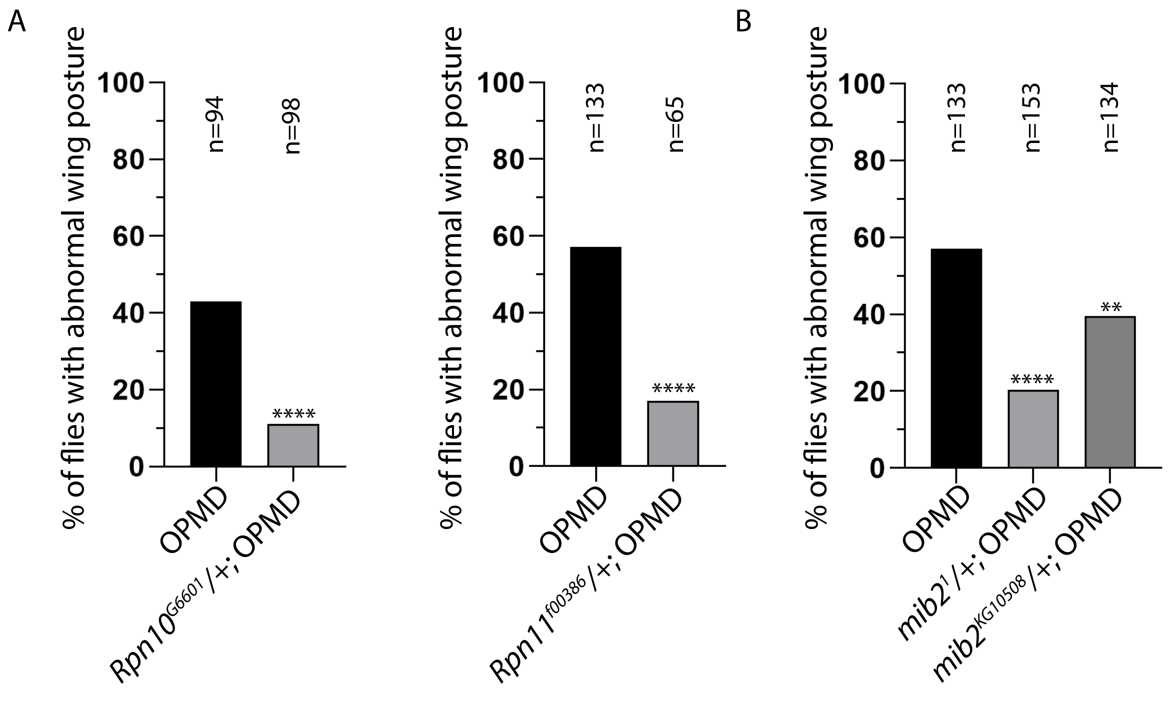

Figure 1 letter: Quantification of OPMD wing position defects in the presence of UPS mutants with different genetic backgrounds.
(A) *Rpn10* and *Rpn11* mutants with different genetic backgrounds from those in Fig. 3B were crossed to *Act88F-PABPN1-17ala* flies. Percentages of flies with abnormal wing position were scored at day 6. The numbers of scored flies are indicated (n). **** p-value <0.0001, using the Chi-square test.
(B) $mib2^{1}$ mutant with a different genetic background from that in Fig. 3B and $mib2^{KG105081}$ were crossed to *Act88F-PABPN1-17ala* flies. Percentages of flies with abnormal wing position were scored at day 6. The numbers of scored flies are indicated (n). **** p-value <0.0001, ** p-value=0.004 using the Chi-square test. $mib2^{1}$ mutant is lethal as L1, whereas $mib2^{KG105081}$ mutant is lethal as L3.
OPMD: *Act88F-PABPN1-17ala/+*.
