# Supplementary material for: Activation of the ubiquitin-proteasome system contributes to oculopharyngeal muscular dystrophy through muscle atrophy
Source: PLoS Genet. 2022 Jan 13;18(1):e1010015. doi: 10.1371/journal.pgen.1010015 (PMC8791501; doi:10.1371/journal.pgen.1010015)
Supplement: Attachment — Submitted filename: Ribot et al.Point-by-point Response.pdf [file pgen.1010015.s013.pdf]

Dr Martine Simonelig
mRNA Regulation and Development
Department Genetics and Development
Institute of Human Genetics, 141, rue de la Cardonille
34396 Montpellier Cedex 5. France
Tel: 334 34 35 99 59
e-mail: Martine.Simonelig@igh.cnrs.fr

PLOS Genetics Editors
PLOS Genetics

Manuscript number: PGENETICS-D-21-00502R1

Montpellier, December 16, 2021

Dear Dr Lu, Dear Dr Barsh,

I thank you very much for your decision regarding our manuscript number PGENETICS-D-21-00502R1, entitled "Activation of the ubiquitin-proteasome system contributes to oculopharyngeal muscular dystrophy through muscle atrophy".

In this new version we have addressed all the remaining points of the reviewers that consisted in textal changes and minor revisions in some of the figures.

The point-by-point response to the reviewers is as follows.

Reviewer #2:

Below are a few minor points that should be attended to for clarity:

Page 11: Regarding the Rpn10 mutant, it is possible that the portion of the transcript that was tested by RT-PCR was produced at normal levels, but the P element in the coding sequence prevented normal translation. This may be what the authors meant by indicating it could be a null allele, but the explanation given in my first sentence would clarify that for the reader.

We have modified the text accordingly p. 11.

Page 11: Explain that the mutant lines reduced the proteins in all tissues, so that is why you did the muscle-specific knockdown.

We have indicated this information on p. 11.

Page 13 top: Change “checked” to “verified”. The word checked does not indicate what the outcome of the experiment was.

We have modified the text accordingly p. 13.

Figure 3E legend: exemplified not examplified (Figure 4C as well). Anti-Kettin appears magenta rather than red in the image I was provided.

We have made these modifications in the legends of Figs. 3 and 4.

Reviewer #3:

Before publication, I would suggest a few more corrections.

- Introduction: extend “we use a Drosophila model of OPMD” to: “we use a Drosophila model of OPMD induced by mesodermal expression of PABPN1” to make clear that it is not a genetic model.

We have modified the text accordingly p. 6.

- Discussion: The authors might elaborate more on the added sentence: “this sequence of events might participate in the progressivity of the disease as proteasome upregulation would be subsequent to defects arising earlier, namely oxidative stress and accumulation of PABPN1 oligomers that might slowly build up

in patients" using, for example, references abundantly quoted earlier in the discussion (23, 24, 65). As it is, extensive description of data from OPMD mouse models is more introduction than discussion of the new data reported here.
We have expanded the Discussion on p. 21.

- New Fig.S1. The figure legend could be more precise and help readers by: i) indicating that 3 adjacent hemisegments are shown. ii) highlighting one specific muscle by its name in the larval panels, (for example LL1 which is well visible and missing in one segment in the right-most panel).
We have added the information that three adjacent hemi-segments are shown in Fig. S1 legend. We have also added a star to show the missing LL1 muscle in Fig. S1B, right panel. However, we have not indicated the name of this specific muscle because we feel that it would be misleading to indicate the name of a single muscle while many other muscles are affected in OPMD larvae.

Reviewer #4:
Interesting work.
In the current manuscript by Ribot et al., the authors have identified the involvement of ubiquitin-proteasome system (UPS) in contribution to oculopharyngeal muscular dystrophy (OPMD) using *Drosophila* as a model system. The authors nicely expand our current knowledge on ubiquitin proteasome system and proposed that proteasome inhibitors would be the potential therapeutic options to treat OPMD. All the experiments were well designed and executed with high standards in the field of *Drosophila* cell biology. Furthermore, I appreciate the authors in making every effort to address previous questions raised by the reviewers and modify the manuscript. Although, the authors have done much work on the revised format, the physiological relevance of these genetic modification is still missing. I would like the authors to incorporate the physiological relevance such as behavioral impact of these genetic manipulation in the manuscript before I am comfortable to provide recommendation that Plos genetics publish this article.
Thank you for this remark that allowed us to clarify this point. We have previously shown that the phenotypes of abnormal wing posture (wings held up or down) that we use to record the suppressor effects of UPS mutants in Fig. 3B reflect both altered muscle function that arises before the appearance of defects in the sarcomeric structure, and muscle degeneration (Chartier et al. EMBO J. 2006, 25, 2253-62). Therefore muscle physiology is recorded through these phenotypes. We have clarified this point in the text p. 11.

Minor comments
1) I would like to suggest the authors to demonstrate fig 2A in figure format.
We believe that for the sake of accuracy it is important to keep all the information present in Fig. 2A, including the FBgn numbers. This is why we maintained this information as a list, as it would be difficult to include all this information in the form of a figure. On the other hand, Fig. 2B is the summary in figure format of the data shown in Fig. 2A.

2) Please put marker in all western blot images. It would be easy to the readers to identify the molecular weight of proteins.
We have indicated the marker sizes in western blots (Figs. 6, 7, S4, S5).

3) Please put scale bars in figure 5A.
We have put scale bars in Fig. 5A.

I hope that you will find this revised version satisfactory. I thank you very much in advance and I am looking forward to hearing from you.

Sincerely yours.

Martine Simonelig
