# Supplementary material for: Activation of the ubiquitin-proteasome system contributes to oculopharyngeal muscular dystrophy through muscle atrophy
Source: PLoS Genet. 2022 Jan 13;18(1):e1010015. doi: 10.1371/journal.pgen.1010015 (PMC8791501; doi:10.1371/journal.pgen.1010015)
Supplement: Attachment — Submitted filename: review Ribot et al., Plos Genetics D-21-00502.docx [file pgen.1010015.s010.docx]

**Plos Genetics D-21-00502**

This ms submitted by C. Ribot et al contains data describing the contribution of increased proteasome activity to oculopharyngeal muscular dystrophy (OPMD). The data are based on an *in vivo*, induced model of OPMD in *Drosophila*, with flight muscles as main model tissue. Specifically, the authors propose that increased proteasome activity, subsequent to (forced) expression and nuclear aggregation of PABPN1-17ala, an OPMD signature, is associated with proteosomal degradation of myofibrillar proteins. This in turn could be causal to muscle defects. Feeding *Drosophila* larvae with a proteasome inhibitor at sub-lethal levels does reduce muscular defects, opening the possibility of future pharmacological treatment of OPMD.

OPMD, a progressive human disease differentially affecting specific sets of muscles is thought to result from accumulation of extended-polyalanine forms of the nuclear PABP PABPN1 (PABPN-11-18ala). Transcriptome analyses previously performed in different models pointed to an increased expression of components of the ubiquitin-proteasome (UPS) protein degradation pathway. The potential involvement of UPS in progression of OPMD has since been an important question, in parallel to mitochondrial dysfunction. Here, the authors address, for the first time in vivo, the consequences of UPS up-regulation using Drosophila flight muscles as model. The starting point is an unbiased genetic screen for suppressors of PABPN17ala-induced lethality, from which the UPS pathway emerges as one candidate pathway (Fig.1-2). The authors assay further the consequences of decreased proteasome activity on induced OPMD, by genetically assaying mutations in 5 individual UPS components. The data show that reduced proteasome activity correlates with reduced PABPN17ala-induced adult muscle defects (Fig 3,4). The rescue does not *consistently* correlate with reduced levels of PABPN1 aggregation, however, although the variability in assays (Fig.5) could prevent firm conclusions (see also Fig6). It correlates with rescue of Mhc and actin levels, compared to OPMD mutants (Fig.6). The authors thus conclude that one consequence of UPS deregulation in PABPN1-17ala mutants is an increased degradation of myofibrillar proteins which could be the molecular basis of OPMD.

An in vivo analysis of the relation between UPS deregulation and OPMD, going from a genetic screen to myofibillar defects, is of interest to a wide range of colleagues. Yet, the data shown are mostly correlative, except for genetic analyses, and rather patchy experiments do not allow strong conclusions. This is well reflected by the general tone of the manuscript with reiteration of “significant” “substantial”, “tendency” terms in author’s conclusions. Below are some specific suggestions for improving the manuscript.

**Comments/suggestions**

Introduction versus discussion

A large part of the introduction recalls diverse molecular consequences of alanine triplet expansion in PAPBPN1, a mutation which leads to PABPN1 aggregates in muscle fibers. The question is then how PABPN1 aggregates lead to progressive weakness of specific muscles. The present ms does little answer this question and ignores the progressive character of the human disease. Many interesting points raised in the introduction need to be better looked at, in the discussion in view of the present data.

Fig. 1. Lethality screen

The screen is based on lethality induced by 24B >PABPN1-17ala, scored in pupae. Although this scoring is adequate for an initial, unbiased screen, a more precise description of the period of lethality and muscle phenotype induced by PABPN1-17ala mesodermal expression should be given. Looking at muscle pattern defects in embryos or larvae, could inform on the selectivity and progression of muscle defects with exercise, and better connect to the rest of the ms (IFM defects).

Fig.3 The adult muscles phenotype

In previous studies, the authors used a muscle specific driver, MhcGal4 (Chartier et al., 2006, 2015). Using this driver, *muscle defects were clear by day 6 and increasing with time to reach 100% of the thoraxes at day 16 (Chartier et al., 2006)*. In this ms, Ribot et al., use Act88FGal4 (ubiquitous expression?) to drive PABPN1-17ala expression in adult IFMs. The fraction of muscle defects (scored at day 11) seems lower. Could the authors comment why they changed driver. Referring to the percentage of muscle defects is very vague and reference to publication [40] for the analysis of these defects seems excessive (see Fig.2 in ref 40, for comparison). As it is, Fig. 4C, the only tissue level analysis within this ms, is not informative. Please indicate the type of defects which were recognized, how muscles were scored as altered, and the changes which occur in either “OPMD” or/and rescued flies between day 6 and 11. It would be a real plus to introduce a sarcomeric marker (see for example Sarov et al., 2016; PMID: 26896675) in order to compare the OPMD phenotype to « myofibrillar genes » phenotypes (Schnorrer et al. ? 2010 PMID: **2022084).** As mentioned for Fig.1, the progressivity, or not, and specificity of the myofibrillar defects deserves more attention.

Fig.4 Proteasome activity

Analysis of proteasome activity is at days 3 and 6 (any change at day 11?) reveals higher activity in OPMD compared to wild type (or Rpn10-rescued) adults, consistent with previous transcriptome analyses. No return to wt proteasome activity levels was observed, however, in transheterozygous flies for 4 of 5 UPS mutants, although rescue of flight muscle/wing posture was effective and similar to Rpn10 (Fig. 3B,3D). Therefore, bases on these sole data, is it legitimate to conclude that *OPMD is associated with increased proteosomal content and activity in muscles that substantially contributes to OPMD* *defects?* Can one exclude that Rpn10 could play other functions?. The lack of consistent correlation does not support fully the conclusion and weakens the rest of the ms.

Fig.5 Reduced OPMD muscle defects by UPS mutants do not correlate with reduced PABPN1aggregation.

The authors observe a correlation between decreased amounts of PABPN1 aggregates and rescue of muscle defects in 3 proteasome mutants (Rpn11, Prosβ4, Pomp). Rpn10 mutants again behaves differently than other UPS mutants, with more aggregates, correlating well with a decreased proteasome activity, as underlined by the authors. Taking into account Fig.4, it seems that the data support the conclusion that rescue of muscle defects does not correlate well with increased proteasome activity, while PABPN1 degradation does (although see Fig.7). The author’s conclusion that « *these data show that the reduction of muscle degeneration in the presence of UPS mutants does not consistently result from reduced PABPN1 aggregation »* is valid but fairly incomplete.

Fig.6 Myofibrillar proteins are more ubiquitinated and degraded in OPMD muscles.

The authors show here that myofibrillar proteins (here, Actin and Mhc) are more ubiquinated and their amounts reduced in OPMD flies. Increased ubiquitination and clearance is already observed at day 3. How do the authors interpret that this reduction does not increase with time, while muscle defects do ?

The myofibrillar proteins amount is restored (strong tendency to --) in some UPS mutant rescued flies (Fig.6E), without, again, consistent correlation with rescue of proteasome activity (Fig.4A, C) or rescue of muscle defects at day 11, Fig. 3D. In view of these results, is it appropriate to conclude that the involvement of the UPS of OPMD pathogenesis depends of its function in the degradation of myofibrillar proteins ?. This may well be true but the data shown are only correlative, and authors need to be more cautious.

Fig.7 Pharmacological inhibition of proteasomal activity reduces OPMD muscle defects

The authors choose the highest non toxic concentration (600 μM) of the proteasomal activity inhibitor MG132 to further assay the role of UPS in OPMD, using wing posture as read-out. Interestingly, “significant” rescue is similarly observed with 400 μM or 500 μM concentrations (Fig. 7B). PABPN1 aggregation is not decreased. These very promising data are consistent with genetic analysis of one UPS mutant, Rpn10, but do not fully explain wing posture rescue by other UPS mutants (Fig.3) which remains very puzzling.

Fig.8

It is worth underlining the new data (in red or bold characters, rather than underlining previous data) and leaving hypothetical connections in italics (for example, degradation (inhibition?) of the PABPN1 oligomer), to make the figure more self-explanatory. If Rpn10 and MG132 to be mentioned in the figure legend, they need to appear on the figure. Muscle atrophy is not what is described here (Fig.4)
