# Supplementary material for: Activation of the ubiquitin-proteasome system contributes to oculopharyngeal muscular dystrophy through muscle atrophy
Source: PLoS Genet. 2022 Jan 13;18(1):e1010015. doi: 10.1371/journal.pgen.1010015 (PMC8791501; doi:10.1371/journal.pgen.1010015)
Supplement: Attachment — Submitted filename: Plos Genetics review_OPMD.docx [file pgen.1010015.s012.docx]

**Activation of the ubiquitin-proteasome system contributes to oculopharyngeal muscular dystrophy through muscle atrophy**

In the current manuscript by Ribot et al., the authors have identified the involvement of ubiquitin-proteasome system (UPS) in contribution to oculopharyngeal muscular dystrophy (OPMD) using *Drosophila* as a model system. The authors nicely expand our current knowledge on ubiquitin proteasome system and proposed that proteasome inhibitors would be the potential therapeutic options to treat OPMD. All the experiments were well designed and executed with high standards in the field of *Drosophila* cell biology. Furthermore, I appreciate the authors in making every effort to address previous questions raised by the reviewers and modify the manuscript. Although, the authors have done much work on the revised format, the physiological relevance of these genetic modification is still missing. I would like the authors to incorporate the physiological relevance such as behavioral impact of these genetic manipulation in the manuscript before I am comfortable to provide recommendation that Plos genetics publish this article.

Minor comments

1) I would like to suggest the authors to demonstrate fig 2A in figure format.
2) Please put marker in all western blot images. It would be easy to the readers to identify the molecular weight of proteins.
3) Please put scale bars in figure 5A.	`
